# The Narghile (Hookah, Shisha, Goza) Epidemic and the Need for Clearing up Confusion and Solving Problems Related with Model Building of Social Situations

**DOI:** 10.1100/tsw.2007.255

**Published:** 2007-10-22

**Authors:** Kamal T. Chaouachi

**Affiliations:** ^1^ Researcher and Consultant in Tobacco Control, Paris, France; ^2^ Teacher of a Comprehensive Course on Hookah, Narghile, Shisha Smoking for French Doctors, University of Paris, XI-XII, Paris, France

**Keywords:** hookah, narghile, shisha, tobacco, smoking, health

## Abstract

Many biomedical studies of the past seven years have failed in giving a sound picture of what hookah (shisha, narghile, goza) smoke and smoking are. The reasons are many: from the widespread use of a confusing neologism (“waterpipe”) instead of the few clear and natural words used for centuries by indigenous and non-indigenous people in their real life, to the use of artificial smoking (machines) instead of relying on quantitative and qualitative analysis of toxicants directly performed on real hookah smokers.

## INTRODUCTION

Further to a recent paper published in this journal by Urkin and colleagues[1], we would like to elaborate a little more on what is now considered a global epidemic that greatly surprised the World Health Organisation[2]. Regrettably, the last decade has witnessed a growing confusion in research on hookah (narghile, shisha, goza) smoking[3]. There are many reasons for this unfortunate situation. To start with, problems have stemmed not only from the linguistic diversity of the studied object but also from a reductionist and nominalist approach that developed through the use of the neologism "waterpipe", a word not found in dictionaries and inexistent in key languages such as Hindi, Urdu, Turkish, Arabic, Hebrew, Persian, etc. or even in scientific English. In contrast, "hookah" and "narghile" can be found in dictionaries and "shisha" has been widely used for ages in the Arab world. Therefore, the use of such a word for all situations, in space and time, is methodologically wrong because the pipe and the smoking products are not the same from one country to the other, from one community to the other, etc. As a simple example, the shisha smoked today by young people in the USA is used with a flavoured tobacco [or no-tobacco]-molasses based mixture called tobamel (tobacco plus "mel" for "honey" in Latin) or mu‘essel (i.e. "honeyed", in Arabic) in Arab countries. Now, this product is completely different from the pure moistened tobacco (called tutun) smoked in a madâ‘a by the Yemeni Jews described by Rakower and Fatal in their famous study[4]. Consequently, extrapolation of findings between both above cases is not appropriate. Unfortunately, this has been done and resulted in frequent bias. As a conclusion, the word

"waterpipe" should be avoided in studies unless extreme and quick simplification is needed. Since contemporaneous hookah smoking bridges between continents and languages, the selection of the right vocabulary remains of utmost important, particularly in questionnaires. Hanna' study is a model in this field[5].

Confusion also appeared when a wrong scenario for the "epidemic" was proposed[3]. The chief reasons behind the world upsurge in hookah smoking were recently summarised in a published table[6]. Apart from the origins of the artefact, which are not Indian, as experts have been repeating for years[3], there has also been some mix-up about many other aspects: underage use, for example[7,8].

## Hookah and Health: Changes

Hookah smoking has not posed any particular medical problem for centuries similarly to the cigar or the short "dry" pipe[9,10]. However, it may today introduce new problems because some of its inner anthropological characteristics have been actually changing over the last two decades. Notably, the nature of the heating source has often changed (from natural to non-natural charcoal) and also the smoking product (from pure tobacco and jurâk to tobamel)[10]. Over the past years, there has also been a great confusion about the conclusions to be drawn from reviews on health effects. In fact, many of the available studies did not distinguish between the different smokers (dependent, not dependent, cigarette smoker, dual cigarette/bidi/hookah smokers, ex-cigarette smoker, etc.) and the smoking products themselves (tumbâk, tobamel, jurâk, etc.)[10]. Fortunately, recent sound studies have focussed on shisha with tobamel (mu‘assel) and their authors have made a remarkable effort, not only in the selection of the volunteers, but also of the important anthropological elements making up the situation (hookah session). Some of them were even carried on in a natural environment (a cafe for instance) and artificial smoking was discarded. This kind of novel research is beginning to shed a useful light on the practice[11,12].

On one hand, the hookah is an excellent filter for dangerous smoke irritants like aldehydes[10] so its smoke is definitely much milder than that from a cigarette. On the other, and because of the charcoal, it generally produces high levels of carbon monoxide[12]. For this reason, it cannot be a valid medical alternative to cigarette use, unlike Swedish SNUS (snuff) for instance. When used heavily (one or more pipes a day), diseases comparable with those caused by cigarettes could appear in the long term (COPD, among others). However, further sound and methodologically rigorous research is needed.

Nicotine levels may vary a lot according to the smoker type and her/his past and present career, the smoking product, the situation, etc. Cohen and colleagues have shown with rats how nicotine might be necessary for the initiation of tobacco dependence but not inevitably for its maintenance[13]. There is in fact a serious debate over the central role of nicotine[14]. Research on the peculiarities of hookah smoking will help reconsider many granted ideas in the study of the general dependence phenomenon caused by cigarette use. Because of "compensation" of the filtered nicotine, ex-cigarette users may be deterred from "switching" to hookah smoking.

Hookah tar is likely to be very different from that produced by cigarettes because of the great differences in temperatures (hundreds of degrees). The evaluation of tar has been a major source of confusion in the world. For many years, any reference to pioneers in this field, like Rakower and Fatal, was dismissed[3,4]. The only findings that have been taken into account for half a decade now were those based on the exploitation of a smoking machine in Lebanon[15,16] despite the recognition that the use of such devices is already not adapted in the field of cigarettes[3,17]. From there, it is logical that in the case of the hookah, where the smoking parameters (number and volume of puffs, interruptions, session length up to ten times that of a cigarette, etc.) are liable to so great changes, the use of such machines should also be avoided.

## Use of Smoking Machines

These studies based on artificial smoking[15,16] did not provide with a realistic picture of the narghile (hookah, shisha) smoker's behaviour, as emphasised in critiques[18] based on deep observations of true smokers[19]. So, the recent idea –of taking into account the unpredictable and non-periodic nature of hookah smoking- put forward six years ago[19], has been eventually adopted to some extent[20]. However, for many and often unexpected reasons, the design of a so-called "playback" narghile smoking machine activated by analog signals[20], instead of binary ones[15,16], is far from being satisfactory.

## AVERAGING

Averaging the behavioural parameters of cigarette users has been a source of a great confusion brought about by the smoking machines based on the FTC (Federal Trade Commission) protocol. As the two former studies by the authors[15,16] were based on a protocol similar in its "philosophy" (air drawn down from a hookah on a periodical basis), a confusion of the same nature was actually transferred to the new field of research on narghile smoking. This was all the more expected that the durations of the respective smoking sessions are completely different.

A second problem is that, according to the FTC protocol, a cigarette is smoked by a machine, not for a given number of puffs but until a certain length of the tobacco rod is burnt. A widely adopted limit is 23 mm from the tip. Indeed, the quantity of toxic substances (carbonaceous material, tar, CO) tends to rise, considerably sometimes, as the burning tip of the cigarette reaches the end or the filter region[21,22,23,24]. In the case of hookah smoking machine, no limit is set for the end of puffing. It is arbitrarily fixed in terms of a questionable number of puffs for the session that, amazingly, vary a lot from one study to the other: 100[15] then 171[16] and 182[20]. Consequently, averaging over such a long period (45-60 min) poses serious methodological problems. Besides, the fact that the charcoal was left for almost one hour in the same position[16] atop the bowl containing the tobamel (mu‘assel) may have contributed in an overproduction of tar and CO. So, some comparisons with cigarettes (as in table 4), are not relevant and even confusing.

## BEHAVIOR

Not only the average number of puffs dramatically increases from one study to the other but also other figures. Volume of puffs switch from 300 ml in 2003 to 530 ml in 2005 to 1020 ml in 2006. Puff duration was 3s in 2003[15], 2.6s in 2005[15] and 3.9s in 2006[20]. Finally, interpuff changed from 30s in 2003 to 17s in 2005 to 15.3s in 2006. The reasons for these variations are not clear at all. Certainly they are based on averaged measures. Unfortunately, averaging in this field entails important biases. If we take puff volumes, leaving aside the question of the bias caused by their periodicity and fixed size[15,16], they vary a lot according to the smoker's career and profile and also the smoked products[25]. People do not smoke the narghile like robots. Their behaviour also depends on the social situations. The authors of the study stated that smokers *"are [normally] engaged in conversation, as well as eating"*[26]. However, this is not reflected at all in the topography. Graphs and tables[20,26] show smokers puffing and dragging all the time. No micro, macro or "breath-taking" event is taken into account: conversation, reading, drinking, sharing the hose, interruptions (changing the charcoal by the café employee every 10 or 15 min), etc. Furthermore, it is assumed that extrapolating this behaviour to the following 30 minutes is demonstrated[26]. For many reasons (nicotine and other substances compensation, flavours, weariness, situation, etc.), we are afraid it is not.

The reference to Morgan[27] is not relevant because the smokers' activity was analysed only once. The 52 anonymous volunteers were apparently all individual smokers. Consequently, they are not representative and selection was biased. The fact that all the volunteers were “willing” to participate[26] actually entails another bias. Unconsciously, they were reminded that they had to smoke. They may have tended to live it up to show “how” they smoke. Some young people even believe that narghile enhances lung capacity[28]. In brief, a smoker who is observed does not behave naturally[19] and even tends to over-ritualise her/his gestures. We actually warned against this methodological trap ten years ago[29].

## TAR

A narghile session needs to have a limit similar to that of cigarettes under the FTC protocol. This limit could be construed as the instant by which the quantity of tar suddenly and abnormally increases because of many factors. One of them is the quantity of tobamel in the bowl and the time the charcoal has been heating it atop the perforated aluminium foil. In the first two studies, the charcoal (self-lighting, not natural; this is a very important point) was kept over the same position for one entire hour[15,16]. The quantity of tobamel (10g) is in great contrast with the average of 20g set in important studies based on a natural environment in Lebanon and Jordan[12,30].

Tar yields obtained with the smoking machine were: 242 mg[15]; 802mg[16]; and 1047mg (DPM)[20]. The smoking mixture may, beyond a certain time, simply get “charred”. This is why waiters in the Middle East coffee-houses change and move the coal around during the session[19]. Strangely enough in the smoking topography[26], they do not. We already stressed that the most renown specialists actually warned against the use of smoking machines in the field of cigarettes[17,31]. This is all the most true for narghile. Consequently, the solution is to measure the intake (CO, tar, etc.) directly on the narghile smoker. For instance, Wu et al.[32] were successful in designing a protocol in which tar yields actually inhaled by human smokers were measured thanks to plethysmograph-based measures coupled with a smoke-trapping device[33] and a vacuum system[34]. As for Adam et al., they have shown that the chemical patterns and smoke components can vary tremendously on a puff to puff basis[35]. Finally, other problems relate to: charcoal (difference laboratory/cafe); separating tobamel/tumbâk smokers; pipes that are not disposable as the FTC protocol would prescribe.

## CONCLUSION

Hookah smoking is a novel though complex field of research. The confusion that has been surrounding its recent revival and upsurge may be cleared up by a greater attention paid to the anthropological context of each situation. The vocabulary should be specific to each of the latter: e.g. hookah, narghile, shisha, madâ‘a, goza, etc. The use of smoking machines should be definitely discontinued and studies in natural environments encouraged.

## COMPETING INTERESTS

No competing commercial interests, but co-inventor on patent application for a no-carbon monoxide harm reduction hookah project (Novagraaf/France). IMPORTANT NOTE: I signed away my past and future rights (total relinquishment) on this harm reduction patent by June 15, 2005, i.e. before its commercial exploitation. From that date, I have not had anymore any relation of any sort with the patent, even if the US patent (Young&Thompson/USA) may still mention my name on the Internet. A legal document was signed on the same date in presence of a State Attorney in Paris (France). I was paid a lump sum, not for the patent, but for my participation in the project.
